# Renal Allograft Pathology Classifications: Contemporary Updates and Diagnostic Utility

**DOI:** 10.7759/cureus.93134

**Published:** 2025-09-24

**Authors:** Hussein Qasim, Hamza Abuuqteish, Karis Khattab, Matteo Luigi Giuseppe Leoni, Giustino Varrassi

**Affiliations:** 1 Pathology and Laboratory Medicine, Jordan University of Science and Technology, Irbid, JOR; 2 Faculty of Medicine, Jordan University of Science and Technology, Irbid, JOR; 3 Medical and Surgical Sciences and Translational Medicine, Sapienza University, Rome, ITA; 4 Pain Medicine, Fondazione Paolo Procacci, Rome, ITA

**Keywords:** antibody-mediated rejection (abmr), banff classification, end-stage renal disease, kidney transplantation, renal allograft pathology, t cell–mediated rejection (tcmr)

## Abstract

Kidney transplantation is the treatment of choice for end-stage renal disease; however, long-term graft survival continues to be jeopardized by rejection, chronic allograft injury, and infection. Histopathological assessment of renal allograft biopsies remains the diagnostic gold standard, yet interpretation variability has historically hindered consistency across institutions and clinical trials. To address this, international consensus efforts established the Banff Classification, with biennial updates refining and expanding its scope. This review outlines the evolution of renal allograft pathology classification systems, emphasizing key Banff revisions that introduced diagnostic criteria for T cell-mediated and antibody-mediated rejection (ABMR), borderline changes, chronic active rejection, and polyomavirus nephropathy. Modern frameworks increasingly incorporate morphology, immunohistochemistry, and emerging molecular diagnostics to improve accuracy and reproducibility. In clinical practice, the Banff system informs immunosuppressive strategies, guides treatment response monitoring, supports prognostication, and standardizes endpoints in clinical trials. Ongoing challenges include interobserver variability, sampling limitations, and restricted access to advanced molecular and digital technologies in resource-limited settings. Future directions point toward integrating multi-omics, digital pathology, and artificial intelligence to create a unified, patient-centered diagnostic platform that extends Banff’s legacy of consensus and adaptability.

## Introduction and background

Kidney transplantation is widely recognized as the optimal treatment for patients with end-stage renal disease, offering improved life expectancy, enhanced quality of life, and reduced healthcare costs compared with long-term dialysis [1]. However, achieving and maintaining long-term allograft survival remains a complex challenge [2]. Immunological rejection, chronic allograft injury, recurrence of the original kidney disease, and infection-related injury can all compromise graft function over time [3]. Histopathological evaluation of renal allograft biopsies remains the gold standard for diagnosing these conditions, despite advances in non-invasive biomarkers and imaging techniques [4]. Through microscopic examination, pathologists can assess the extent and type of tissue injury, differentiate between immune-mediated and non-immune causes of graft dysfunction, and identify features of chronic damage that influence prognosis [5]. However, interpreting allograft biopsies is inherently complex [6,7]. Without standardized definitions and grading thresholds, diagnostic variability can occur not only between different institutions but also among individual pathologists [8]. Such inconsistencies have historically hindered the comparability of research studies, reduced the reliability of clinical trial endpoints, and occasionally led to misaligned clinical management strategies [9]. Recognizing these challenges, the transplant community developed formal classification systems for renal allograft pathology [6]. The most influential of these, the Banff Classification, emerged in 1991 from a unique international collaboration between pathologists, nephrologists, transplant surgeons, and immunologists [10]. Its consensus-based structure and iterative updates have allowed it to adapt to evolving scientific understanding while remaining a globally accepted reference standard [11]. This narrative review aims to provide a comprehensive account of the classification systems used in renal allograft pathology. Special emphasis is placed on the Banff Classification, including its historical development, the rationale for major revisions, and its current structure. Complementary systems, such as Kidney Disease: Improving Global Outcomes (KDIGO) recommendations and institution-specific adaptations, are discussed alongside practical applications in routine patient care. The review also addresses ongoing limitations, such as interobserver variability and the integration of molecular tools, and concludes by exploring future directions that may redefine graft pathology in the coming decades.

## Review

Methods

This narrative review was conducted to synthesize the evolution, clinical utility, and limitations of renal allograft pathology classifications. Although not a systematic review, steps were taken to ensure transparency and rigor in the literature selection process. A comprehensive search of PubMed, Excerpta Medica Database (Embase), Scopus, and Google Scholar was performed. Reference lists of relevant articles and consensus documents were also screened manually to identify additional publications. The search included literature published between January 1991, coinciding with the first Banff meeting, and July 2025. This range was selected to capture the full history of the Banff Classification and related developments. Search strategies combined free-text terms and Medical Subject Headings (MeSH), including “renal allograft pathology,” “Banff Classification,” “kidney transplant rejection,” “antibody-mediated rejection,” “transplant biopsy,” “digital pathology,” and “molecular diagnostics.” Boolean operators (AND/OR) were applied to optimize results. Consensus conference reports (e.g., Banff, KDIGO), original research studies, narrative and systematic reviews, and authoritative nephrology and pathology textbooks were considered eligible. Conference abstracts without full data, case reports, and non-English language publications were excluded. Only articles published in English were included. This decision was based on the global predominance of English-language consensus reports and to ensure accurate interpretation of diagnostic terminology. The included studies were grouped and narratively synthesized under five major domains: historical evolution of renal allograft pathology classification; current frameworks and diagnostic categories; clinical applications in transplant practice; challenges and limitations; and future directions, including molecular and digital innovations. Evidence was not pooled statistically but rather summarized qualitatively to highlight consensus themes, advances, and persisting gaps.

Historical overview of renal allograft pathology classification

The history of renal allograft pathology classification reflects the broader evolution of kidney transplantation itself [12]. In the early decades of transplantation, particularly during the 1960s and 1970s, biopsy interpretation was largely descriptive and heavily dependent on the experience of the individual pathologist [13]. Diagnostic terminology such as “acute rejection” or “chronic rejection” was applied without agreed-upon histologic thresholds, and the criteria for diagnosing rejection could vary substantially from one center to another [14]. Clinical management decisions were often guided by a combination of physician experience, patient symptoms, and laboratory values, with the biopsy report serving as an interpretive rather than standardized tool [15]. By the late 1980s, the rapid growth of transplant programs worldwide, coupled with the increasing complexity of immunosuppressive regimens, made the need for a uniform pathological classification more urgent [16]. The absence of standardized criteria created several problems [17]. Interobserver variability was a major challenge, as pathologists in different institutions could arrive at different diagnoses from the same biopsy specimen [18]. Research efforts were similarly hindered, since multicenter trials lacked a consistent pathology endpoint, making it difficult to compare outcomes or pool data across studies [19]. Communication between clinicians and pathologists was also affected, with variability in reporting contributing to inconsistent treatment strategies for similar pathological findings [20]. The first Banff Conference on allograft pathology, held in 1991 in Banff, Alberta, Canada, marked a significant turning point [10]. This landmark meeting gathered experts from multiple disciplines (pathology, nephrology, surgery, and immunology) to create a consensus-based classification that would be applicable across institutions and adaptable over time [10]. The result was the original Banff Classification, published shortly after the meeting, which established a set of defined histologic lesions relevant to rejection and other allograft pathologies, introduced a semi-quantitative scoring system for each lesion, and created composite diagnostic categories derived from these scores to enable reproducible diagnosis of acute and chronic rejection [10]. From the outset, the Banff Classification was conceived as a living document to be revised periodically through subsequent biennial meetings [10,21]. This structure allowed it to incorporate new scientific insights, emerging diagnostic tools, and clinical feedback, ensuring that the system evolved alongside advances in transplantation medicine [22]. Over the following decades, these revisions expanded the scope of the classification from an initial focus on acute T-cell-mediated rejection to include antibody-mediated rejection, borderline changes, chronic active rejection, polyomavirus nephropathy, and other entities of clinical importance [23]. While the Banff system quickly became the dominant global standard, other frameworks also contributed to the field [24]. The KDIGO initiative issued guidelines that incorporated Banff criteria into broader clinical practice recommendations, thereby reinforcing its role in patient management [25]. In some high-volume transplant centers, local modifications to Banff scoring were introduced to align with institutional protocols or research priorities [26]. However, these adaptations rarely displaced Banff as the central international reference standard [26]. The historical development of renal allograft pathology classification is, therefore, a story of international collaboration, iterative refinement, and the recognition that pathology must serve both research standardization and immediate clinical decision-making [6]. The ongoing evolution of these systems reflects the dynamic nature of transplant medicine and the continuous pursuit of more precise, reproducible, and clinically relevant diagnostic frameworks [6].

Current classification systems

The Banff Classification

The Banff Classification provides a systematic approach to the diagnosis of renal allograft pathology [10]. It is built upon a series of defined histologic lesions, each graded semi-quantitatively on a scale, typically from 0 to 3, based on severity [10]. These individual lesion scores are combined according to established algorithms to generate an integrated diagnosis [27]. This process reduces subjectivity, improves reproducibility, and facilitates communication between pathologists and clinicians [27].

**Figure 1:**
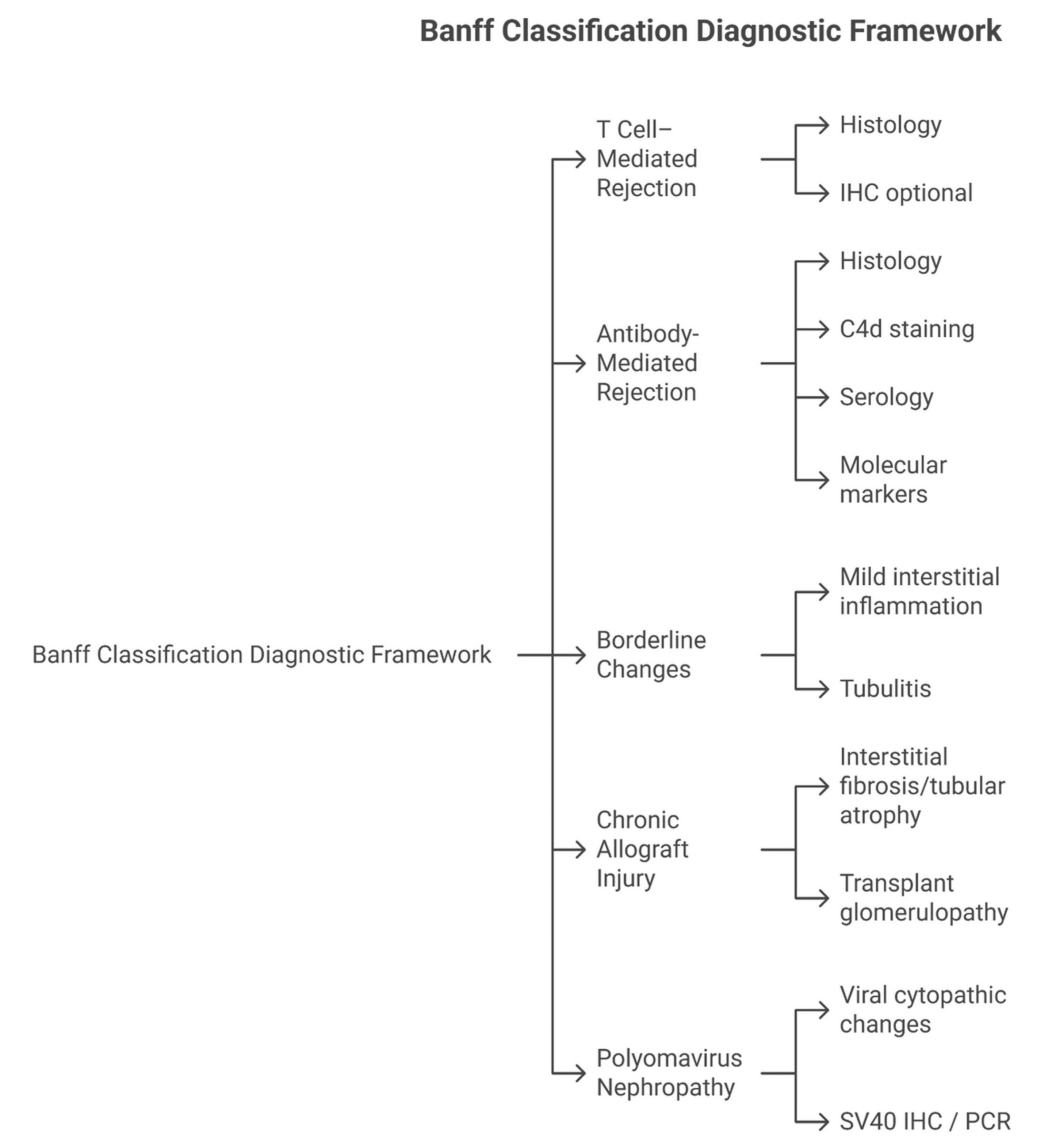
Diagnostic categories in the Banff Classification. Image created by Dr. Karis Khattab on Microsoft PowerPoint (Microsoft Corporation, Redmond, Washington, United States)

Within this framework, several major diagnostic categories are recognized, each with specific histologic, immunohistochemical, and, in recent versions, molecular criteria [28]. T-cell-mediated rejection (TCMR), formerly referred to as acute cellular rejection, is characterized by interstitial inflammation and tubulitis [29]. Lesion scoring focuses on the degree of interstitial inflammation (i-score), the severity of tubulitis (t-score), and, when present, vascular involvement (v-score) [30]. The combination of these scores determines the grade of TCMR, which can range from borderline changes to severe vascular rejection [30]. In addition, antibody-mediated rejection (ABMR) is defined by evidence of tissue injury in the microvasculature, such as glomerulitis (g-score) and peritubular capillaritis (ptc-score), along with evidence of antibody interaction with the endothelium, most notably C4d deposition, and serologic evidence of donor-specific antibodies (DSA) [31]. Over time, the Banff criteria have expanded to recognize C4d-negative ABMR and to incorporate molecular markers, reflecting the evolving understanding of the humoral rejection mechanism [32]. Furthermore, borderline changes are applied when findings suggest the possibility of TCMR but fall short of established thresholds [33]. This category is important in clinical practice because it may signal early or mild rejection that could progress if untreated [34]. Importantly, incomplete humoral rejection lesions lacking both C4d deposition and DSA should also be categorized, as they may represent an early or atypical manifestation of antibody-mediated injury [34]. Chronic allograft injury, initially described under the broad term “chronic allograft nephropathy,” has evolved into more specific descriptors such as interstitial fibrosis (ci-score) and tubular atrophy (ct-score) [35]. Other chronic changes, including transplant glomerulopathy (cg score) and chronic vascular lesions (cv score, ah score), are also scored, as these features are critical for prognostication and assessing cumulative graft injury over time [36]. Moreover, polyomavirus nephropathy, recognized as a distinct category in later Banff updates, is graded based on the extent of viral cytopathic changes and the degree of interstitial fibrosis and tubular atrophy [37]. Its inclusion highlights the importance of distinguishing infection-related injury from immune-mediated rejection [37]. Each Banff category integrates morphological observations with ancillary studies [27]. Immunohistochemistry for C4d, SV40, or BK virus antigens, as well as electron microscopy and, increasingly, molecular diagnostics, are incorporated into diagnostic algorithms [38]. This multimodal approach reflects the complexity of allograft injury and the need for layered evidence to achieve diagnostic certainty [39]. The Banff Classification provides a structured framework for categorizing renal allograft pathology. The major diagnostic categories and their key criteria are summarized in Figure 1.

Other Classification

The KDIGO initiative provides international clinical practice guidelines for the care of kidney transplant recipients [40]. While KDIGO does not propose an independent histopathological classification, it explicitly endorses the use of the Banff criteria for biopsy interpretation [40]. KDIGO guidelines emphasize the importance of biopsy-proven diagnoses before initiating or escalating immunosuppressive therapy, highlighting the role of standardized pathology in avoiding unnecessary or harmful treatments [40]. KDIGO also provides guidance on when to perform biopsies, how to interpret results in the context of clinical and laboratory data, and how to integrate pathological findings into patient management plans [41]. This linkage between pathology and clinical decision-making reinforces the practical utility of the Banff framework, ensuring that histologic diagnoses are not viewed in isolation but are interpreted in a multidisciplinary context [42]. Some high-volume transplant centers have developed localized adaptations of the Banff criteria [42]. These modifications may include institution-specific cutoffs for lesion scores, the integration of center-specific molecular tests, or customized reporting templates to align with local electronic medical record systems [10]. In certain research settings, expanded scoring systems are used to capture subtle changes that might not meet formal Banff thresholds but are considered important for longitudinal studies [43]. While these adaptations can enhance internal consistency and facilitate research, they are generally designed to remain compatible with Banff terminology to preserve comparability across institutions [12]. In recent years, advances in molecular diagnostics have opened new frontiers in renal allograft classification [44]. Transcriptomic analysis of biopsy tissue, for example, can identify gene expression profiles associated with TCMR or ABMR, even in cases where morphological changes are subtle or equivocal [45]. Other emerging approaches include proteomic and metabolomic profiling, which have the potential to identify novel biomarkers of graft injury, and digital pathology, which enables quantitative morphometric analysis and facilitates artificial intelligence-assisted diagnosis [45]. While these methods are not yet universally available, they represent the future trajectory of renal allograft pathology classification, promising greater precision and earlier detection of injury.

Major updates in the Banff Classification

**Figure 2:**
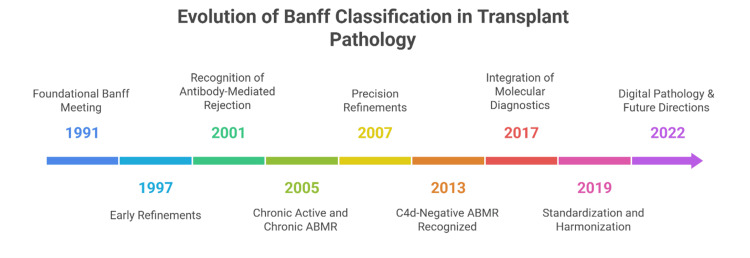
Evolution of the Banff Classification of renal allograft pathology (1991–2022). Image created by Dr. Karis Khattab on Microsoft PowerPoint (Microsoft Corporation, Redmond, Washington, United States) ABMR: antibody-mediated rejection

One of the defining features of the Banff Classification is its deliberate design as a “living” system [6]. As shown in Figure 2, this classification has gone through various stages of updates and refinements.

From its inception in 1991, the framework has been revised at biennial consensus conferences to incorporate advances in transplant immunology, pathology, and diagnostic technology [10]. These updates are not cosmetic adjustments; rather, they represent meaningful refinements that respond to emerging evidence, clarify diagnostic thresholds, and address areas of clinical uncertainty [46]. Over time, these revisions have both broadened and deepened the classification, transforming it from a primarily histology-based system into a multidimensional diagnostic platform that integrates morphology, immunohistochemistry, and molecular markers [47]. Table 1 illustrates the key changes, rationale, and clinical impact of each update of the Banff Classification. 

**Table 1:** Key changes, rationale, and clinical impact of each update of the Banff Classification. TCMR: T-cell-mediated rejection; ABMR: antibody-mediated rejection; KDIGO: Kidney Disease: Improving Global Outcomes; DSA: donor-specific antibodies

Year	Key Changes	Rationale	Clinical Impact
1991 [10]	Introduction of the Banff Classification; lesion-based semi-quantitative scoring (i, t, v); composite diagnoses for acute rejection; chronic changes grouped under “chronic allograft nephropathy.”	Establish an internationally standardized framework for renal allograft biopsy interpretation.	Provided a common diagnostic language, enabling reproducibility, multicenter research comparability, and improved clinician–pathologist communication.
1997 [48]	Refined chronic lesion scoring (ci, ct); clarified borderline changes category.	Differentiate chronic injury patterns and recognize early/subtle immune-mediated changes.	Allowed better prognostication; prompted closer clinical follow-up for borderline cases.
2001 [49]	Formal recognition of antibody-mediated rejection (ABMR) with diagnostic triad: microvascular injury, C4d positivity, donor-specific antibodies (DSA).	Growing evidence of humoral rejection as a major cause of graft loss; availability of C4d staining.	Transformed biopsy workup with routine C4d immunostaining; led to targeted therapies for ABMR.
2005 [50]	Inclusion of chronic active and chronic ABMR; recognition of transplant glomerulopathy (cg) and peritubular capillary basement membrane multilamination as chronic ABMR features.	Evidence that antibody-mediated injury can be chronic and progressive.	Improved recognition of late graft injury patterns; informed long-term immunosuppression strategies.
2007 [51]​	Stricter criteria for borderline TCMR; emphasis on microvascular inflammation scoring (g + ptc).	Reduce overdiagnosis of borderline cases; improve diagnostic precision for ABMR.	Decreased unnecessary immunosuppression; enhanced detection of clinically significant ABMR.
2013 [51]​​​​​​​​	Recognition of C4d-negative ABMR; acceptance of molecular markers as supportive evidence.	Studies showed ABMR can occur without C4d but with DSA and endothelial gene expression signatures.	Expanded ABMR diagnosis; increased detection of cases previously missed; guided treatment of C4d-negative rejection.
2017 [52]​​​​​	Formal integration of molecular diagnostics into Banff criteria; refined chronic active TCMR definitions.	Evidence that molecular profiles improve diagnostic accuracy and prognostic value.	Encouraged adoption of molecular testing; improved recognition of active immune injury in fibrotic tissue.
2019 [53]​​​​​​​	Standardization of lesion definitions; harmonization with KDIGO and clinical trial endpoints; guidance on integrating molecular data.	Reduce interobserver variability; align with broader transplant care frameworks.	Increased diagnostic consistency; facilitated data sharing and trial design.
2022 [54]​	Introduction of concepts for digital pathology, AI-assisted morphometry; reinforced clinical–pathology integration; future-focused molecular integration.	Anticipation of technological shifts in pathology; need for scalable, precise diagnostic tools.	Set groundwork for AI and multi-omics use; positioned Banff to incorporate next-generation diagnostics.

Banff 1991: The Foundational Framework

The original Banff meeting in 1991 produced the first internationally agreed-upon histopathologic classification for renal allograft biopsies [10]. The initial framework concentrated on what was then termed acute cellular rejection, currently referred to as TCMR, and established a semi-quantitative scoring system to grade key histologic lesions such as interstitial inflammation (i-score), tubulitis (t-score), and vascular intimal arteritis (v-score). Chronic changes were encompassed under the umbrella term “chronic allograft nephropathy,” without further subclassification. Importantly, this first iteration established the principles of lesion-based scoring and composite diagnoses, which remain at the heart of the Banff system today.

Banff 1997: Early Refinements and Chronic Lesions

By the late 1990s, clinical experience revealed the limitations of the original chronic allograft nephropathy designation, which grouped diverse chronic injuries under a single label [48]. The 1997 update began to refine chronic injury descriptors, introducing more specific scoring for interstitial fibrosis (ci) and tubular atrophy (ct). The meeting also clarified criteria for borderline changes, cases with mild interstitial inflammation and tubulitis that fell short of diagnostic thresholds for acute rejection but often carried clinical significance.

Banff 2001: Recognition of Antibody-Mediated Rejection

The early 2000s marked a paradigm shift in transplant pathology with the formal recognition of ABMR as a distinct pathological entity [49]. Although humoral rejection had been suspected for decades, it was the demonstration of peritubular capillary C4d deposition as a marker of complement activation that provided a reproducible histologic correlate. The 2001 Banff update established the diagnostic triad for ABMR: morphologic evidence of microvascular injury, immunopathologic evidence of antibody interaction with the endothelium (usually C4d positivity), and serologic evidence of DSA. This addition transformed clinical practice by prompting routine C4d staining of allograft biopsies and changing the therapeutic approach to suspected rejection episodes.

Banff 2005: Incorporation of Chronic ABMR

The 2005 meeting expanded the ABMR category to include chronic active and chronic forms [50]. Transplant glomerulopathy (cg lesions) and multilamination of peritubular capillary basement membranes on electron microscopy were recognized as characteristic chronic ABMR features. This broadened perspective acknowledged that antibody-mediated injury could persist at low levels for years, producing slowly progressive graft dysfunction that was often resistant to therapy.

Banff 2007: Greater Precision and Borderline Clarification

By 2007, clinicians and pathologists were seeking more precise thresholds for certain categories, especially borderline TCMR [51]. The update refined criteria for borderline changes, reducing overdiagnosis by requiring at least mild tubulitis in multiple tubules. In ABMR, additional emphasis was placed on microvascular inflammation scores, encouraging a more integrated assessment of glomerulitis (g) and peritubular capillaritis (ptc) rather than relying solely on C4d staining.

Banff 2013: Recognition of C4d-Negative ABMR

One of the most influential revisions occurred in 2013, with the recognition of C4d-negative ABMR [51]. Clinical and molecular studies have shown that some cases of microvascular injury with circulating DSA lacked detectable C4d deposition but still demonstrated poor graft outcomes. The updated criteria allowed for ABMR diagnosis in such cases, provided there was other evidence of antibody-endothelial interaction, such as validated molecular signatures. This change was particularly significant because it expanded the scope of ABMR diagnosis and highlighted the limitations of relying on a single biomarker.

Banff 2017: Integration of Molecular Diagnostics

The 2017 update represented a turning point toward molecular pathology. Gene expression profiling, especially transcripts associated with endothelial injury, was formally recognized as supportive evidence for ABMR diagnosis [52]. The meeting also refined chronic active TCMR criteria, addressing cases with ongoing inflammation in areas of interstitial fibrosis and tubular atrophy. These refinements acknowledged that chronic rejection could be an active, immune-mediated process rather than a static scar.

Banff 2019: Standardization and Harmonization

The 2019 revision placed emphasis on standardizing lesion scoring and improving reproducibility across centers [53]. Definitions of key lesions, such as intimal arteritis and microvascular inflammation, were further clarified. The update also provided more explicit guidance on integrating molecular data into routine diagnosis and discussed approaches for harmonizing Banff criteria with KDIGO recommendations and clinical trial endpoints. This version highlighted the need for ongoing education and training to reduce interobserver variability.

Banff 2022: Digital Pathology and Future Directions

The most recent Banff update, in 2022, addressed emerging trends in digital pathology, artificial intelligence, and automated image analysis [54]. While still largely histology-centered, the framework began to envision a future in which digital morphometry could enhance scoring precision and molecular data could be seamlessly integrated into diagnostic reports. The classification also reinforced the importance of contextualizing biopsy findings within the patient’s clinical history, laboratory values, and immunologic profile. Discussions at this meeting underscored that the Banff system must remain flexible to accommodate rapidly advancing technologies.

The most recent Banff consensus (2022-2023) also provided additional refinements to the diagnostic criteria for ABMR, emphasizing microvascular inflammation scoring and integrating serologic/molecular evidence to improve reproducibility [54]. Chronic active TCMR definitions were also updated, recognizing the clinical impact of persistent inflammation in fibrotic tissue. These refinements reflect an ongoing shift toward more granular and clinically meaningful categorization of allograft injury [55,56].

Practical applications in clinical practice

The Banff Classification, while developed as a histopathological framework, has far-reaching implications in the clinical management of renal transplant recipients [50]. Its structured lesion scoring and diagnostic categories provide more than a purely academic exercise; they serve as a critical bridge between microscopic findings and therapeutic decision-making [55]. In practice, the classification is applied not only to confirm or rule out rejection but also to tailor immunosuppression, evaluate treatment response, determine prognosis, and standardize outcomes in research and clinical trials [56]. Perhaps the most direct application of the Banff Classification lies in its influence on immunosuppressive management [27]. A diagnosis of acute TCMR, for example, typically prompts intensification of corticosteroid therapy, consideration of antithymocyte globulin for higher-grade lesions, and adjustments in maintenance immunosuppressive regimens [57]. Similarly, identification of ABMR can lead to targeted interventions such as plasmapheresis, intravenous immunoglobulin, rituximab, or complement inhibitors [58]. By grading lesions on a semi-quantitative scale, the Banff criteria help clinicians distinguish between mild, moderate, and severe forms of rejection [42]. This granularity allows for proportional therapeutic responses, avoiding overtreatment of borderline changes while ensuring aggressive intervention for severe or rapidly progressing rejection [59]. In some centers, specific Banff scores are directly embedded into clinical algorithms, ensuring that therapeutic decisions are both evidence-based and standardized [32]. The classification also plays a pivotal role in prognostication [32]. Chronic changes such as interstitial fibrosis (ci-score), tubular atrophy (ct-score), transplant glomerulopathy (cg-score), and chronic vascular changes (cv-score) are associated with poorer long-term outcomes and reduced graft survival [60]. Quantifying these lesions provides a measure of cumulative injury, enabling clinicians to counsel patients about long-term expectations and to identify those who may benefit from closer surveillance or enrollment in clinical trials for novel therapies [61].

**Figure 3:**
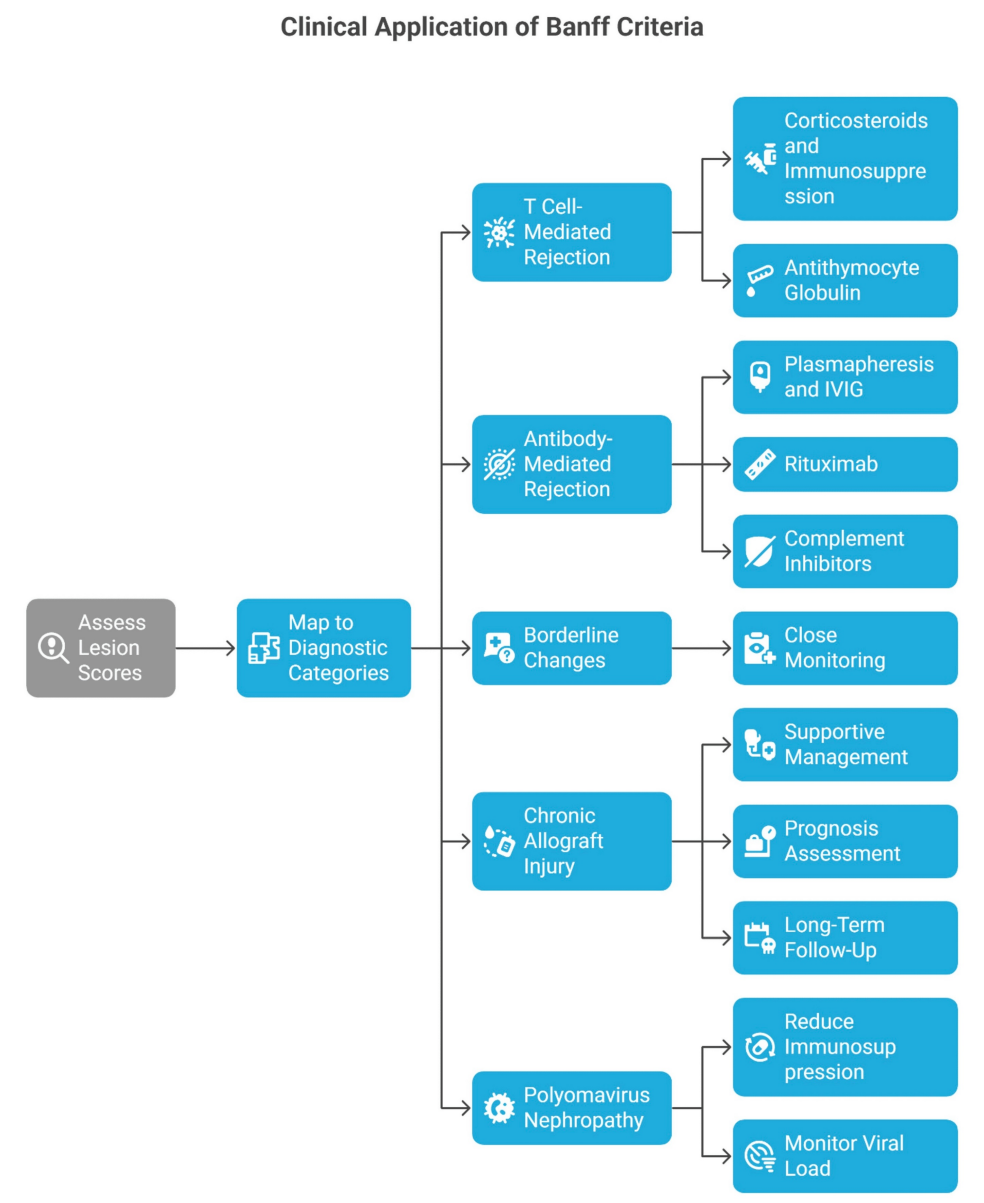
Clinical applications of the Banff criteria. Image created by Dr. Karis Khattab on Microsoft PowerPoint (Microsoft Corporation, Redmond, Washington, United States)

Importantly, the integration of acute and chronic lesion scores in a single biopsy allows for nuanced interpretation [62]. For example, chronic injury with superimposed active inflammation may carry a worse prognosis than finding it alone, prompting more urgent therapeutic intervention [63]. Serial allograft biopsies interpreted through the Banff framework are used to monitor the effectiveness of anti-rejection therapy [27]. A decrease in inflammatory lesion scores after treatment can confirm therapeutic efficacy, whereas persistence or worsening of lesions may indicate treatment resistance, ongoing immune activation, or the emergence of alternative causes of injury such as drug toxicity or infection [64]. In research settings, standardized Banff scoring is invaluable for evaluating new immunosuppressive agents, as it allows for reproducible measurement of histologic response across trial sites and time points [65]. Clinical trials in transplantation require objective, reproducible endpoints to compare therapeutic interventions [66]. Banff lesion scores and diagnostic categories provide such endpoints, enabling multicenter studies to collect comparable histopathological data [67]. This standardization has facilitated numerous landmark trials investigating immunosuppressive strategies, tolerance-inducing protocols, and therapies for ABMR [68]. Without a consensus classification, the variability in biopsy interpretation across centers would significantly undermine the reliability of such research [69]. The Banff Classification serves as a shared language between pathologists, nephrologists, transplant surgeons, and other members of the transplant team [70]. By providing explicit lesion scores and diagnostic categories, pathology reports become more transparent and actionable [71]. For example, instead of a vague diagnosis such as “mild rejection,” a Banff-based report might read: “Interstitial inflammation score 2, tubulitis score 1, meeting criteria for Banff grade IA TCMR.” [72] This precision allows clinicians to understand the exact nature and severity of the pathology and to tailor management accordingly [72]. While rejection remains a central concern in allograft pathology, many cases of graft dysfunction are due to non-immune causes, including drug toxicity (e.g., calcineurin inhibitor nephrotoxicity), recurrent or de novo glomerular disease, infection (such as polyomavirus nephropathy), and vascular complications [73]. Banff-based assessment helps differentiate these conditions by providing specific morphological criteria [6]. This distinction is critical because the management of non-immune injury often involves reducing immunosuppression rather than increasing it, a decision that, if made incorrectly, could jeopardize graft survival [74]. In patients with failing grafts, the pathology report can influence decisions about re-transplantation eligibility and timing [75]. For instance, the presence of severe chronic ABMR with ongoing DSA positivity may prompt consideration of desensitization strategies before listing for another transplant [76]. Conversely, a graft failing from non-immunologic causes, with minimal sensitization, might predict a more favorable outcome with a subsequent transplant [77]. The Banff Classification not only provides a diagnostic framework but also guides therapeutic decisions in renal transplant pathology. Figure 3 illustrates the clinical decision pathway from lesion scoring to diagnostic categorization and subsequent management strategies.

Challenges and limitations

Despite its widespread adoption and continuous refinement, the Banff Classification and renal allograft pathology more broadly face several enduring challenges that can affect diagnostic accuracy, reproducibility, and clinical applicability [78]. These limitations arise from the intrinsic complexities of allograft pathology, technical aspects of biopsy sampling, and the broader realities of healthcare delivery across different settings [79]. One of the most frequently cited concerns is interobserver variability among pathologists [80]. Even with explicit lesion definitions and scoring criteria, the subjective interpretation of histological changes can lead to discrepancies [81]. Lesions such as mild interstitial inflammation, subtle tubulitis, or low-grade microvascular inflammation are particularly prone to differing interpretations [82]. Studies have shown that diagnostic agreement is highest for clear-cut cases of severe rejection but lower for borderline or minimal changes [83]. This variability has implications for both clinical care and research, as inconsistent diagnoses can lead to inappropriate treatment escalation or undertreatment and can reduce the comparability of multicenter trial data [84]. Efforts to mitigate this variability include standardized training programs, use of reference image libraries, and inter-laboratory quality control exercises [85-87]. The potential application of digital pathology and artificial intelligence-assisted image analysis offers promise for improving consistency in lesion scoring, though these technologies are still being validated for widespread use [88]. The diagnostic accuracy of a biopsy depends heavily on the adequacy of the sample [89]. Banff guidelines recommend a minimum of seven glomeruli and one artery for a biopsy to be considered adequate for rejection assessment [90]. Inadequate sampling may lead to false-negative results, particularly for focal lesions such as vascular rejection or localized microvascular inflammation [91]. In some cases, repeat biopsies may be required, which exposes patients to additional procedural risks and delays diagnosis [92]. Sampling adequacy can be influenced by technical factors, such as needle gauge, number of passes, and operator experience, as well as by patient factors, including obesity, coagulopathy, and anatomic variations [93]. Some centers have adopted real-time ultrasound guidance and on-site adequacy assessment by a pathologist or trained technologist to improve diagnostic yield [94,95]. Similarly, chronic injury patterns such as interstitial fibrosis and tubular atrophy (IF/TA) can result from multiple mechanisms, including immune-mediated damage, drug toxicity, recurrent or de novo glomerular disease, and donor-derived pathology [96,97]. While the Banff framework encourages this integrative approach, it cannot entirely eliminate diagnostic uncertainty in cases with overlapping features [55]. Although recent Banff revisions have incorporated molecular diagnostics as supportive evidence, access to such testing is uneven across institutions and countries [98]. Molecular assays for endothelial injury transcripts or other rejection-associated gene profiles can improve diagnostic accuracy, especially in cases of C4d-negative ABMR, but they require specialized equipment, trained personnel, and additional cost [99]. In resource-limited settings, reliance on morphology and basic immunohistochemistry may limit the sensitivity of diagnosis, particularly for subtle or early rejection [100]. The Banff Classification is designed for universal applicability, but not all centers have the same diagnostic infrastructure. Routine C4d immunostaining, electron microscopy, or molecular assays may be unavailable in many parts of the world [101]. Even when available, financial constraints or reimbursement policies may restrict their use [102]. In such settings, pathologists may need to rely on modified diagnostic algorithms, potentially leading to under-recognition of certain rejection types and impacting patient outcomes [103]. While the iterative nature of the Banff Classification is a strength, it also presents challenges for clinicians who must adapt to new criteria and nomenclature [103]. Revisions that expand or contract diagnostic categories can affect reported rejection rates, influence treatment thresholds, and alter longitudinal data interpretation [104]. This is particularly relevant in clinical trials, where protocol-defined endpoints may change mid-study if an update is adopted.

Future directions

The Banff Classification will continue to evolve alongside advances in transplant immunology, pathology, and diagnostic technology [6]. Future developments are likely to focus on integrating molecular, digital, and non-invasive approaches to improve precision and accessibility [105-107]. Beyond current transcriptomic applications, combining genomics, proteomics, and metabolomics with histology could enable earlier and more accurate detection of allograft injury, as well as insights into underlying mechanisms for targeted therapy [108]. Whole-slide imaging and AI-assisted morphometry offer opportunities to standardize lesion scoring, reduce interobserver variability, and detect subtle histological changes [109]. Such tools may be especially valuable where experienced transplant pathologists are scarce. Biomarkers such as donor-derived cell-free DNA, urinary chemokines, and exosomal RNA could complement or reduce the need for routine biopsies, reserving invasive procedures for cases where non-invasive testing suggests injury [110,111]. Future classifications may incorporate individualized immune profiles and pharmacogenomic data to guide therapy selection, dosing, and intensity, minimizing unnecessary immunosuppression while targeting high-risk patients [112]. Moreover, diagnostic frameworks, open-access digital resources, and standardized training can help ensure that advances are equitably implemented, including in resource-limited settings [113]. Multi-center validation efforts, such as Halloran and colleagues’ Molecular Microscope Diagnostic System (MMDx) studies, have demonstrated the utility of transcriptomic signatures in diagnosing and stratifying rejection phenotypes across diverse cohorts [114]. More recent NGS-based approaches confirm the reproducibility of molecular diagnostics, reinforcing their potential as complementary or alternative tools to morphology in future Banff iterations [115].

## Conclusions

Over the past three decades, the Banff Classification has revolutionized renal allograft pathology, evolving from a primarily descriptive discipline into a standardized, reproducible, and clinically relevant framework. Its iterative revisions reflect the rapidly advancing field of transplantation medicine, incorporating novel pathological entities, refining diagnostic criteria, and integrating innovations in immunohistochemistry, molecular diagnostics, and, more recently, digital pathology. By establishing a universal language for pathologists, nephrologists, and transplant surgeons, the classification enhances diagnostic technology, informs treatment decisions, and enables consistent reporting across institutions and clinical studies. Its influence extends beyond histopathology, shaping immunosuppressive regimens, guiding prognosis, and harmonizing research outcomes. Despite its impact, challenges persist, including interobserver variability, limitations in biopsy sampling, overlapping histologic features, and disparities in access to advanced diagnostic technologies. Looking ahead, the future of renal allograft pathology lies in integrating histologic, molecular, and computational data into a patient-centered diagnostic platform, one that promises earlier injury detection, improved risk stratification, and personalized therapy. This approach holds promise for earlier detection of injury, improved risk stratification, and personalized therapy. Upholding Banff’s tradition of transparency, consensus, and adaptability will be key to ensuring these advancements translate into better graft survival and patient outcomes worldwide.
